# An effective method for refining predicted protein complexes based on protein activity and the mechanism of protein complex formation

**DOI:** 10.1186/1752-0509-7-28

**Published:** 2013-03-28

**Authors:** Jianxin Wang, Xiaoqing Peng, Qianghua Xiao, Min Li, Yi Pan

**Affiliations:** 1 School of Information Science and Engineering, Central South University, Changsha 410083, China; 2 Department of Computer Science, Georgia State University, Atlanta, GA 30302-4110, USA

**Keywords:** Protein activity, Protein complex formation model, Just-in-time, Refining, Gene expression

## Abstract

**Background:**

Identifying protein complexes from protein-protein interaction network is fundamental for understanding the mechanism of cellular component and protein function. At present, many methods to identify protein complexes are mainly based on the topological characteristics or the functional similarity features, neglecting the fact that proteins must be in their active forms to interact with others and the formation of protein complex is following a just-in-time mechanism.

**Results:**

This paper firstly presents a protein complex formation model based on the just-in-time mechanism. By investigating known protein complexes combined with gene expression data, we find that most protein complexes can be formed in continuous time points, and the average overlapping rate of the known complexes during the formation is large. A method is proposed to refine the protein complexes predicted by clustering algorithms based on the protein complex formation model and the properties of known protein complexes. After refinement, the number of known complexes that are matched by predicted complexes, *Sensitivity*, *Specificity*, and *f*-measure are significantly improved, when compared with those of the original predicted complexes.

**Conclusion:**

The refining method can discard the spurious proteins by protein activity and generate new complexes by just-in-time assemble mechanism, which can enhance the ability to predict complex.

## Background

In a cell, rather than function individually or in isolation, proteins interact physically with each other to form multisubunit protein complexes that act as sophisticated multimolecular machines, such as the anaphase-promoting complexes, RNA splicing and polyadenylation machinery, protein export and transport complexes[1]. The functionality of the cell depends on protein physical interactions and these multimolecular machines, thus great effort has been made to identify and describe all protein-protein interactions (PPIs) and protein complexes in a cell. The maturity of high-throughput experimental techniques and computational prediction, such as two-hybrid assay, mass spectrometry experiments, and the protein chip technology, make it possible to construct large-scale protein-protein interaction networks (PPINs) of many species. Many protein complexes are well understood, particularly in the model organism Saccharomyces cerevisiae (a strain of yeast). For this relatively simple organism, the study of protein complexes is now being performed genome wide and the elucidation of most protein complexes of the yeast is undergoing. Predicting protein complex from protein interaction networks is one of the most challenges but a fundament to analyze tissue and protein functionality [2-4]. Graph theory has become a powerful research tool for analyzing PPINs [5]. In graphs which are used to represent PPINs, proteins are represented by vertexes or nodes, and PPIs are represented by edges. Computation methods based on graph theory are also used to predict protein complex [6].

So far, many clustering methods are developed for identifying proteins complexes in PPINs [2-5,7-28]. Dense sub-graph based methods and hierarchy algorithms are two representative categories. Based on the assumption that the members in the same protein complex and functional module strongly bind each other, a cluster can be referred as a densely connected subgraph within a PPIN. The density (*d*) of a subgraph with *n* vertices and *m* edges is generally defined as *d*=2*m*/(*n*(*n*−1)) [9]. Maximal cliques are used in several algorithms to identify protein complexes [9,14,22,24,29]. Spirin et al. [9] employ *d* = 1 to identify the maximal cliques as protein complexes. An algorithm named Clique Percolation Method (CPM) [29] is proposed by uniting the maximal cliques with *k*−1 common nodes, and the well-known protein network analysis tools CFinder [14] is developed on it. The maximal cliques in CMC [22] are generated from a weighted PPIN, and then combined or removed based on connectivity and overlapping rate. Wang et al [24] propose a new topological model by extending the definition of *k*-clique community of algorithm CPM and introducing distance restriction, and develop a novel algorithm called CP-DR based on the new topological model to identify protein complexes. Some other dense sub-graph based complex detection algorithms follow a “seed and extension” paradigm, such as MCODE [30], Density-Periphery based graph clustering algorithm (DPClus) [16], IPCA [4], and Core-Attachment method [21], using different mechanisms of seeds selection, cluster expansion and stop conditions to detect protein complexes. Recently, entropy-based graph clustering methods are also applied on PPINs to detect dense sub-graphs as protein complexes [27,28]. Hierarchical clustering algorithms are based on similarity or distance to identify protein complexes, with the idea that the majority of proteins within a same protein complex tend to have similar or identical functions [31]. The similarity or distance between any two proteins is defined as the possibility of the two proteins in the same functional module. The most classic hierarchical clustering method is GN algorithm [8]. MoNet algorithm [19] is a typical coagulation, derived from the GN algorithm. HCS algorithm [32] is used to analyze the protein modular structure based on graph connectivity. HC-PIN method [26] uses the weighted edge clustering coefficient to perform fast hierarchical clustering.

Besides the topological characteristics or the functional similarity features of protein complexes, some researchers try to reveal the mechanism of protein complex formation. Focusing on the dynamic formation of protein complexes, De Lichtenberg et al. [33] construct time-dependent PPIN of Yeast by integrating PPIN and gene expression data. By analyzing the dynamics of protein complexes during the yeast cell cycle, they discover that most complexes are constituted by both periodically and constitutively expressed proteins, which suggests a mechanism of just-in-time assembly. Based on gene expression data, Komurov and White [34] also classify proteins of eukaryotic into periodically expressed proteins and constitutively expressed proteins. However, by analyzing the topology of the two classes of proteins, they find that most functional modules are consisted by proteins from one class (periodically expressed proteins or constitutively expressed proteins), seldom from both of the two classes. The different conclusions drawn from De Lichtenberg and Komurov might be caused by the difference of data sources and the considered module types.

A protein is active when it is in its active form, and it can interact with other active proteins and perform function [35]. During the formation of a complex, at one time point the co-active proteins will assemble together, and at the next time point the new co-active proteins will be added in, therefore the complex can be assembled step by step following a just-in-time way. In this paper, a protein complex formation model is presented based on the just-in-time mechanism. Relied on the protein complex formation model and combined with gene expression data, an investigation is carried out on 408 known complexes of yeast [36] and the protein complexes identified by existing methods. Based on the protein complex formation model and the properties of known complexes, we propose an effective method to refine the complexes predicted by existing methods. Several clustering algorithms are applied on yeast PPIN to predict protein complexes. The *speciality*, *sensitivity*, *f*-measure and other evaluation metrics are compared between the original predictions and the refined predictions.

## Methods

In this section, we first introduce our former study on protein activity, which is deduced from gene expression data. Later on, based on the active time points of proteins and the protein complex formation model, we investigate the formation of each known complex. These analyses are also carried out on the complexes predicted by existing methods. Based on the difference between known complexes and predicted complexes, an effective method is introduced to refine the complexes predicted by existing methods.

### Material

The PPI data of yeast is downloaded from DIP [37] updated to Feb. 28, 2012. The Database of Interacting Proteins (DIP) is a database that provides species specific subsets, which contains all the interactions of proteins from a particular species. In our experiment, the self-interactions and repeated ones in the original PPIN are discarded. The final PPIN used in our experiment contains 5023 proteins and 22570 interactions.

We use GSE3431 [38] in GEO to extract active time points of each protein, which is an expression profile of yeast by array affymetrix gene expression data over three successive metabolic cycles. The overall design of this expression experiment is 12 time intervals per cycle, and 25 minutes per time interval. Thus each gene has gene expression values (levels) at 12 time points in each cycle. In our method, one cycle with average expression value at every time point of three successive cycles is used to reduce the noise and error.

### Protein active information

A protein is active when it is in its active form, and it can interact with other active proteins and perform functions. The activity of a protein not only can be affected by its surrounding environment, but also can be regulated by controlling its amount and lifetime in the cell [35]. In our previous study[39], we focus on the latter to deduce protein activity. The control may be exercised at several places in the flow of information from genes to proteins. At simplest, the amount of a protein can be set by the level of transcription, which in turn can be controlled by, for example, the strength of the promoter or the action of a transcription factor, which may be a repressor or activator. The mRNA level may also be adjusted after transcription by varying the rate of RNA degradation. At the protein level, quantities are controlled by the lifetime of the molecule, which is determined by its rate of degradation. The rate of turnover varies considerably from proteins to proteins. There are several specific mechanisms for targeting protein molecules to the cell degradation machinery, including covalent attachment of the small protein ubiquitin. Thus each protein has its active periods, and we assume a protein is active at the time points with its highest expression level. Because the expression level of a protein will be decreased after the protein has completed its function that leads a feedback for controlling the expression quantity, while its rate of turnover is constant. In our previous study, we propose a 3-sigma principle [39] to differentiate the inactive and active points of a protein during a cellular cycle by combining gene expression data. The 3-sigma strategy is to design an active threshold for each gene by considering its own characteristic expression curve and the inevitable noise in gene expression array. A harmonic threshold for a protein *p* based on its algorithmic mean and variance can be calculated.

(1) $$u\left(p\right)=\frac{\overset{n}{\underset{i=1}{\sum }}EV_{i}(p)}{n}$$

(2) $$\sigma^{2}\left(p\right)=\frac{\overset{n}{\underset{i=1}{\sum }}{\left(EV_{i}\left(p\right)-u\left(p\right)\right)}^{2}}{n}$$

(3) $$F\left(p\right)=\frac{1}{1+\sigma^{2}\left(p\right)}$$

(4) $$\text{Active}\text{\_}\text{Th}\left(p\right)=S_{1}\left(p\right)\times F\left(p\right)+S_{2}\left(p\right)\times \left(1-F\left(p\right)\right)$$

In Equations (1) and (2), *n* is the number of time points in a cell cycle, and *EV*_*i*_(*p*) is the expression value of *p* at time point *i*. In Equation (4), *S*_1_(*p*)=*u*(*p*) and *S*_2_(*p*)=*u*(*p*)+3*σ*(*p*). The detail about the active threshold principle (3-sigma principle) is presented in [39].

For each gene product *p*, *u*(*p*) is the algorithmic mean of its expression values and *σ*(*p*) is the standard variance of its expression values. *F*(*p*) reflects the fluctuation of its expression curve. The higher Standard Variance, and the smaller the *F*(*p*). As shown in Equation (4), the active threshold of *p* is determined by both algorithmic mean and 3-sigma. If the fluctuation of expression values is low, corresponding to small *σ*(*p*), *S*_1_(*p*) plays a more important influence on the active threshold. Reversely, *S*_2_(*p*) plays a more important role to determine the active threshold. Protein *p* is considered as active at some time points only when the expression values of these time points are above its active threshold *Active*_*TH*(*p*). *Active*(*p*) is an active time point set of protein *p*, which contains the time points when protein *p* is active, defined as Equation 5.

(5) $$\;\text{Active}(p)=\left\{i\left|i\in \left[0\ldots 12\right],EV_{i}(p)\geq \text{Active}\text{\_}\text{TH}(p),p\in V\right)\right\}$$

In our experiments, although 96% of the proteins in the yeast PPIN from DIP can be covered by gene products in this gene expressing profile, the active time points of a small portion of proteins cannot be deduced from this gene expression data. The active information of 1142 proteins cannot be inferred from their expression curves in this gene expression profile, and 177 proteins have no expression values in this gene expression data. These proteins might be active in other gene expression experiments, since the interval between two time points in GSE3431 is considerably long and some proteins are active only under special environments. If the active time points of protein *p* cannot be inferred from this gene expression profile, a special active time point “0” is used. That is to say *Active* (*p*)={0} in this case.

### Protein complex formation mechanism and protein activity

According to the just-in-time mechanism, a complex *C* can be formed in a continuous time point set. A complex formation model based on the just-in-time mechanism can be illustrated as follows. Suppose *AP*_*i*_(*C*) (*i*=0,…,*n*) is the set of proteins which are active at time point *i* and belong to the complex *C*, where *n* is the number of time points in a cell cycle. *C* can be formed in a continuous time point set [*S*,*S*+*K*], if the boolean function *FC*(*C*,*S*,*K*) is true. If the follow conditions are satisfied, *FC*(*C*,*S*,*K*) is true, otherwise it is false. 

(1)

$$\left|\left(\overset{S+j}{\underset{i=S}{⋃}}AP_{i}(C))\cap AP_{S+j+1}(C\right)\right|>0$$,

for *j*=0,…,*K*−1

(2)

$$\left|\overset{S+K}{\underset{i=S}{⋃}}AP_{i}(C)\right|=\left|C\right|$$

If we can find a interval [*S*,*S*+*K*] for complex *C* that can make *FC*(*C*,*S*,*K*) be true, we say that the complex can be formed in the continuous active time point set. If there exists no interval for complex *C* that can make *FC*(*C*,*S*,*K*) be true, we say that the complex cannot be formed in a continuous active time point set. Combined with the active time point set of each protein and this model, a statistic is carried on the 408 known protein complexes of Yeast [36] and the complexes predicted by representative algorithms to calculate the percentage of complexes which can be formed in a continuous active time point set. The comparison is listed in Table 1. About 63.4% known complexes can be formed in a continuous time point set, while only a small portion of predicted complexes can be formed in a continuous time point set.

**Table 1:** The comparison of average co-active rate and the percentage of complexes formed in a continuous time point set of the known complexes and complexes predicted by algorithms

**Complexes**	**Average**	**Formed**
	**( *CoActiveRate *)**	**complexes(%)**
Known Complexes	0.595	63.4%
Predicted Complexes(CMC)	0.210	23.0%
Predicted Complexes(DPClus)	0.329	36.5%
Predicted Complexes(IPCA)	0.269	29.3%
Predicted Complexes(CPM)	0.335	37.0%
Predicted Complexes(MCL)	0.252	28.8%
Predicted Complexes(Core)	0.265	29.7%

The average co-active rates of the known complexes and the complexes predicted by six algorithms are compared in Table 1. The percentages of complexes which are formed in a continuous time point set in the known complexes and predicted complexes are list in Table 1.

For complex *C*, when *FC*(*C*,*S*,*K*) is true, the overlapping rate of time point *S*+*i* is the fraction of the number of common members in *AP*_*S*+*i*_(*C*) and the protein set assembled in time points [*S*, *S*+*i*−1] to the minimum size of two sets, defined as Equation (6). The overlapping of complex *C* during the formation interval [*S*,*S*+*K*] is the average of the overlapping rates of time points from *S*+1 to *S*+*K*, defined as Equation (7). The average overlapping of the known complexes is calculated, so do the complexes predicted by representative algorithms, as shown in Table 1. We can observe that the average overlapping of the known complexes is above 0.5, and that of the complexes predicted by each algorithm is significantly lower. Therefore the property that protein complexes are formed step by step is neglected and the predicted complexes contain spurious proteins. Based on these shortcomings, we can refine the predicted complexes.

(6) $$OL_{S+i}(C)=\frac{\left|\left(\overset{S+i-1}{\underset{h=S}{⋃}}AP_{h}(C))\cap AP_{S+i}(C\right)\right|}{\text{Min}(\left|\left(\overset{S+i-1}{\underset{h=S}{⋃}}AP_{h}(C)\right)\right|,AP_{S+i}(C))},i=1,\mathrm{..},K$$

(7) $$\text{OL}(C)=\frac{\overset{K}{\underset{i=1}{\sum }}OL_{S+i}(C)}{K}$$

### Refining method

To improve the accuracy of complex prediction, we propose a method to refine the protein complexes predicted by existing methods based on protein activity and the protein complex formation model with the just-in-time mechanism.

#### The framework of the method

**Figure 1:**
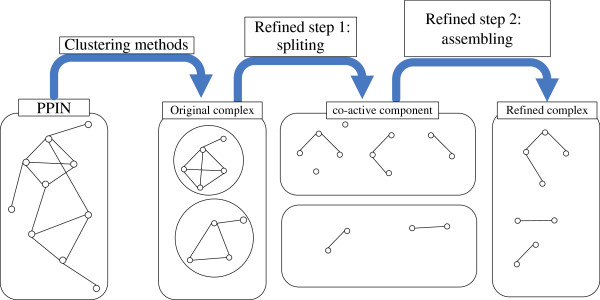
**The outline of the refinement.** The refining method has two steps, including splitting and assembling. In the framework of the refinement, a clustering method is firstly applied on a PPIN to predict protein complexes, and the splitting and assembling processes are consecutively carried on each predicted complex.

The active proteins of a predicted complex at each time point can form several sub-clusters based on PPIN topology. The overlapping between two clusters, labeled as *OV*(*C*_*i*,*k*_, *C*_*j*,*l*_), is defined as the fraction of the number of common members in the two clusters to the minimum size of two clusters, shown as Equation (8), where *C*_*i*,*k*_ is a sub-cluster *k* at time point *i*, and *C*_*j*,*l*_ is a sub-cluster *l* at time point *j*. Based on the overlapping of sub-clusters at adjacent time points, a protein complex can be formed step by step. Only the complexes, whose size is greater than 2, will be refined, because the complex with size=2 has only two possible forms after refinement, two singletons or the same complex with no change. The refining method has two steps, including splitting and assembling. The overall framework of the refining method is shown in Figure 1. Firstly, a clustering method is applied on a PPIN to predict complexes. Then, the splitting and assembling processes are consecutively carried out on each predicted complex.

(8) $$\text{OV}(C_{i,k},C_{j,l})=\frac{\left|C_{i,k}\cap C_{j,l}\right|}{\text{Min}(\left|C_{i,k}\right|,\left|C_{j,l}\right|)}$$

#### Splitting of protein complex

The active time set of a complex, defined in Equation (9), is constituted by the active time points of each protein in the complex. During the splitting, an original predicted complex can be split according to *n*+1 time points, where *n* is the number of time points in a gene expression profile and an additional special time point ‘0’ is used to contain the proteins whose *Active* (*p*)={0}. For each active time point, the proteins which are not active at the time point are excluded, thus the complex can be split into several connected components (sub-clusters) at the time point. In each component, all proteins are active at the time point.

(9) $$\text{Active}\text{\_}C(C_{i})=\left\{t\left|t\in \text{Active}(v),v\in C_{i}\right)\right\}$$

The detail of the splitting algorithm is shown in Algorithm 1. The inputs of this algorithm are an original complex which contains proteins and interactions derived from PPIN, and the active time point set of the complex. For each active time point, the proteins which are not active at this time point will be removed from the original complex, and several sub-clusters are generated based on PPIN topology.

### Algorithm 1 Splitting

After splitting, a complex will become many sub-clusters on its whole active time point set. Most sub-clusters are smaller than the original complex. Some sub-clusters may be singletons, some sub-clusters may be identical ones, and some ones are high-overlapping with each other.

#### Assembling of protein complex

Based on the just-in-time mechanism, the assembling of a protein complex is to assemble the sub-clusters with certain overlapping at adjacent time points. The core idea of assembling is to combine the sub-clusters from adjacent time points that satisfy *OV*(*C*_*i*,*k*_,*C*_*i*+1,*l*_)≥*T*, where *T* is a threshold to discriminate high overlapping from low overlapping. Active time point ‘0’ is used to denote a special active time point for proteins whose active time points cannot be inferred from the current gene expression profile. These proteins have the potential to be active at some time points when special conditions are prepared. Thus, the sub-clusters at ‘0’ time point can combine with sub-clusters at arbitrary time points with overlapping great than *T*.

The description of assembling algorithm is shown in Algorithm 2. Firstly, let the sub-clusters at time point ‘0’ combine with sub-clusters at other time points. If there exists *C*_*i*,*k*_ at time point *i* and *C*_0,*l*_ at time point ‘0’ satisfying *OV*(*C*_0,*l*_,*C*_*i*,*k*_)≥*T*, let *C*_*i*,*k*_ be the union of *C*_0,*l*_ and itself. After all sub-clusters at each time point have been checked, delete the sub-clusters in the set at time point ‘0’ which have been combined. Secondly, the sub-clusters at time point *i* and the adjacent time point *i*+1 are checked. For each *C*_*i*,*k*_ at time point *i*, if there exists *C*_*i*+1,*l*_ that satisfies *OV*(*C*_*i*+1,*l*_,*C*_*i*,*k*_)≥*T*, let *C*_*i*+1,*l*_ be the union of *C*_*i*,*k*_ and itself. After all the sub-clusters in the set at time point *i* set have been checked, the sub-clusters at time point *i* are deleted if they have been combined. Finally, the left sub-clusters at each time point are considered as new protein complexes. These new protein complexes are assembled according to the time order, connectivity and the overlapping of active proteins. After the assembling process, an original protein complex might become several new protein complexes, or be discarded. New protein complexes tend to be smaller than the original ones. After all original predicted protein complexes are undertaken the splitting and assembling steps, the identical ones in the final results are deleted.

### Algorithm 2 Assembling

glbb.eps" Format="JPEG" Color="Color" Type="LinedrawHalftone" Rendition="HTML"/>

**Figure 2:**
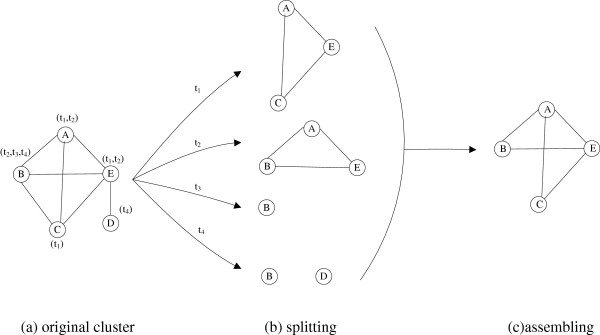
**An example of refinement.** Figure 2 shows the refinement on an original predicted complex, composed by five proteins, A, B, C, D, and E. Figure 2(**b**) are sub-clusters at each time point set after the splitting. By combing the high-overlapping sub-clusters in adjacent time points, a new protein complex is generated as shown in Figure 2(**c**).

The refinement process of an original complex is illustrated in Figure 2. As shown in Figure 2(a), an original complex is composed by five proteins, A, B, C, D, and E. Figure 2(b) shows sub-clusters at each time point set after the splitting. For example, at time point “ *t*_1_”, protein A, C and E are active and connective with each other, thus they can form a sub-cluster. Protein A, B and E are active at time point “ *t*_2_” and form a sub-cluster. At time point “ *t*_3_”, only protein B is active. At time point “ *t*_4_”, protein B and D are active, but not connective with each other, thus each of them becomes singletons. By combining the high-overlapping sub-clusters in adjacent time points, a new protein complex is generated, which contains A, B, C, and E, as shown in Figure 2(c).

### Evaluation metrics

In earlier studies [16,26,30], the overlapping score *OS*(*Pc*, *Kc*), shown in Equation (10) is used to assess how effectively a predicted complex *Pc* matches a known complex *Kc*.

(10) $$\text{OS}(\text{Pc},\text{Kc})=\frac{|V_{\text{Pc}}\cap V_{\text{Kc}}{|}^{2}}{\left|V_{\text{Pc}}|\times |V_{\text{Kc}}\right|}$$

where |*V*_*pc*_| is the number of proteins in the predicted complex and |*V*_*Kc*_| is the number of proteins in the known complex. If a predicted complex *Pc* that has no common proteins with a known complex *Kc*, then *OS*(*Pc*, *Kc*)=0. Usually, a predicted complex and a known complex are considered as a match if their overlapping *OS* is no less than 0.2 [16,26,30]. If we say a predicted complex *Pc* perfectly matches a known complex *Kc*, it means all proteins appears in *Pc* are equal with that in *Kc*, and *OS*(*Pc*, *Kc*)=1. *PC* is the number of the predicted complexes. *MKC* is the number of known complexes that are matched by predicted complexes and *MPC* is the number of predicted complexes that match the known complexes, when a threshold value of *OS* is adopted. *MKC* illustrates the ability to predict complex.

Sensitivity (*Sn*) is the fraction of the known complexes that are matched by the predicted complexes (*OS*≥0.2) among all the known complexes [30], shown in Equation (11). Specificity (*Sp*) is the fraction of the predicted complexes that match the known complexes (*OS*≥0.2) to the total number of the predicted complexes [30], defined in Equation (12).

(11) $$\text{Sn}=\frac{\text{TP}}{\text{TP}+\text{FN}}$$

(12) $$\text{Sp}=\frac{\text{TP}}{\text{TP}+\text{FP}}$$

where *TP* (True Positive) is the number of the predicted complexes that match the known complexes (*OS*(*Pc*, *Kc*) ≥ 0.2), *FP* (False Positive) is the number of the predicted complexes that don’t match the known complexes, and *FN* (False Negative) is the number of the known complexes that are not matched by any predicted complexes.

*f*-measure combines *Sn* and *Sp*[30], defined in Equation (13).

(13) $$f-\text{measure}=\frac{2\times \text{Sp}\times \text{Sn}}{\text{Sp}+\text{Sn}}$$

## Results and discussion

In order to evaluate the efficiency of the refining method, we apply it to refine the protein complexes predicted by six representative clustering algorithms. DPClus and IPCA are density-based local search algorithms [16,26]. Clique Percolation Method (CPM) is a powerful algorithm to find protein complexes[29] and MCL is a fast and highly scalable clustering algorithm for networks based on stochastic flow [40,41]. CMC [22] and Core-Attachment [21] are the latest ones for detecting community structures. We use the parameters recommended by their authors in these algorithms.

In the experiments, the size of predicted complexes which need to be refined should be not smaller than 3. According to the analysis of known complexes, the average co-active rate of the known complexes is above 0.5, thus in our refined method the *OV*’s threshold *T* is set as 0.6. The impact of the varying of *T* on the accuracy of protein complexes prediction is analyzed in subsection “Analysis of parameter *T*”.

### Comparison with known complexes

For convenience sake, the complexes predicted by each clustering method are mentioned as original complexes, the complexes refined by our method are referred as refined complexes, denoted as algorithm_O and algorithm_R in tables and figures, respectively. The original complexes and refined complexes of each algorithm are compared with the known protein complexes obtained from the literature published in Nucleic Acids Research [36] separately. There are 408 manually annotated complexes which are considered as the gold standard data and of which each consists of two or more proteins.

To evaluate the performance of the refining method on the original complexes with different sizes, we select six algorithms, three of which are good at identifying relatively small complexes and the others are good at predicting relatively large complexes. Table 2 lists the numbers and average sizes of the original complexes and refined ones of each algorithm. The number of predicted complexes of each method is increased after refinement, and the average size of refined complexes is smaller than that of the original ones, because the refining method filters some non co-actived proteins out of the original complexes, and reassembles the co-active proteins into new complexes based on the just-in-time mechanism. The average sizes of original complexes of MCL, Core-Attachment, and CPM are relatively large, and after refinement the average sizes are larger than 3. However, the average size of refined complexes of CPM is bigger than that of the original ones. The reason is that an original super-complex with 1821 proteins predicted by CPM becomes many large-size sub-clusters after splitting, while these large sub-clusters are so hard to satisfy the overlapping threshold (*T*=0.6) that they cannot be combined with each other. The average sizes of the original complexes predicted by CMC, IPCA and DPClus are relatively small, and after refinement the average sizes of the refined complexes are smaller than 3.

**Table 2:** The average size of the complexes predicted by algorithms before and after refinement

**Algorithm**	***PC***	**Average Size**
CMC_O	1369	3.45
CMC_R	1532	2.48
DPClus_O	383	3.98
DPClus_R	578	2.76
IPCA_O	1650	3.72
IPCA_R	1843	2.67
CPM_O	197	13.30
CPM_R	346	17.48
MCL_O	621	6.71
MCL_R	877	3.62
Core_O	675	6.10
Core_R	1025	3.49

The complexes of each clustering method before and after refinement are denoted as algorithm_O and algorithm_R. Table 1 lists the numbers and average sizes of the original complexes and refined ones of each algorithm.

**Figure 3:**
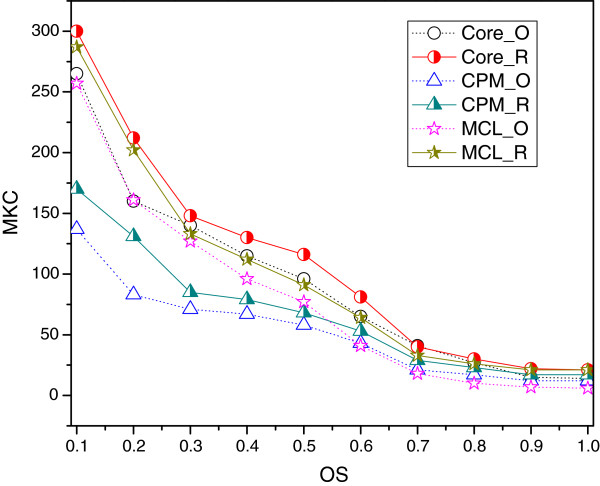
**The *MKC * of CPM, MCL and CoreAttachment under different *OS * threshold values.** The complexes of each clustering method before and after refinement are denoted as algorithm_O and algorithm_R. The *MKC* before and after refinement of CPM, MCL and CoreAttachment algorithms are compared with respect to different *OS* threshold values.

For the algorithms which predict complexes with larger average sizes, from Figure 3, it can be found that the *MKC* of three algorithms after refinement are improved significantly under different *OS* threshold values, compared with those of the original complexes. It demonstrates that the refining method can discard the spurious proteins by protein activity and generate new complexes by just-in-time assemble mechanism, which can enhance the ability to predict complex.

**Figure 4:**
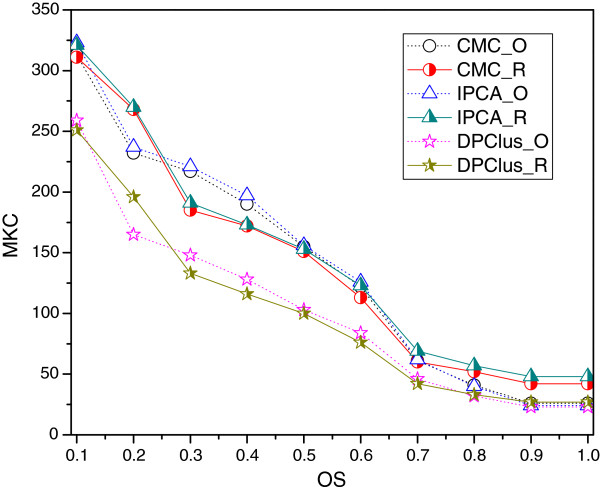
**The *MKC * of CMC, IPCA and DPClus before and under different OS threshold values.** The complexes of each clustering method before and after refinement are denoted as algorithm_O and algorithm_R. The *MKC* before and after refinement of CMC, IPCA and DPClus algorithms are compared with respect to different *OS* threshold values.

In Figure 4, *MKC* before and after refinement of the algorithms with relatively small average sizes of predicted complexes are compared with respect to different *OS* threshold values. When the value of the *OS* threshold is changed from 0.7 to 1, *MKC* after refined is improved. Because these three methods prefer to find small complexes, and the average sizes of the refined complexes are smaller than 3, which makes the overlapping score between the refined complexes and the known complexes mainly fall in [0.7, 1], in which *MKC* will increase after refinement. Therefore, for the algorithms that predict relatively small complexes, the refining method can make the prediction more precise.

**Figure 5:**
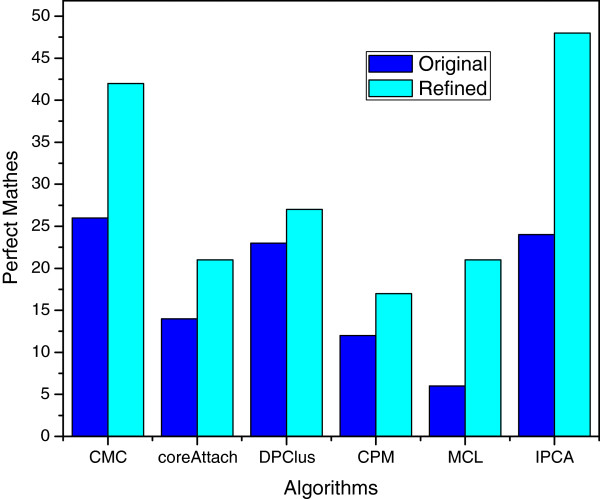
**The numbers of perfect matching ( *OS *=1) of six algorithms before and after refinement.** Figure 5 shows the numbers of perfect matching from the original predicted complexes and the refined complexes of CPM, MCL, CoreAttachment, CMC, IPCA and DPClus algorithms.

Since the spurious proteins of original complexes at each time point are discarded by the refining method, a more precise prediction is supposed to be available for each algorithm. In Figure 5, we can observe that the numbers of perfect matches are increased after refinement, and the average gain of perfect matches of six algorithms is 12. The number of perfect matches of CMC_R is 42, which is 16 more than that of CMC_O. 24 more perfect matches are gained by IPCA_R, while the number of perfect matches of IPCA_O is 24. MCL_R has 15 more perfect matches, compared with 6 perfect matches gained by MCL_O. DPClus_O has 23 perfect matches, while DPClus_R has 27 perfect matches. CoreAttachment_R gains 7 more perfect matches compared with that of CoreAttachment_O. The CPM_R has 17 perfect matches, and CPM_O has 12 perfect mathes.

### Analysis of sensitivity, specificity and *f*-measure

**Figure 6:**
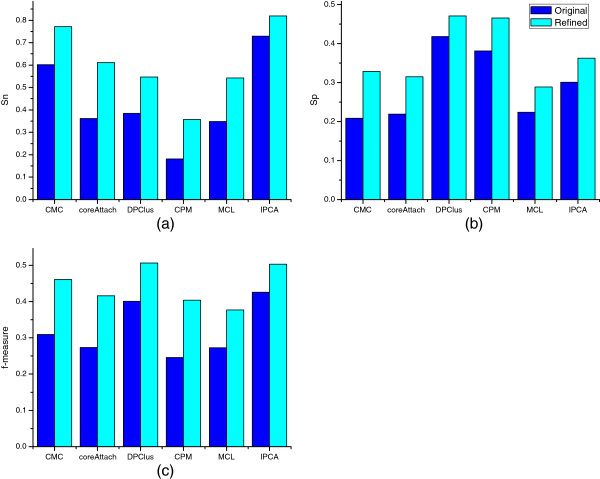
**The comparison of *f *-measure, *Sn *, *Sp * of six algorithms before and after refinement.** (**a**) is the comparison of *Sensitivity*(*Sn*) of the complex predictions of six algorithms before and after refinement; (**b**) is the comparison of *Specificity*(*Sp*) of the complex predictions of six algorithms before and after refinement; (**c**) is the comparison of *f*-measure of the complex predictions of six algorithms before and after refinement.

In Figure 6(a), the *Sn* of refined complexes of each algorithm is higher than that of the original ones. The improvements of *Sn* of most algorithms are above 15%, except for IPCA, which is about 9%. The potential ability to identify more known complexes is available in each algorithm. The room for the improvement of *Sn* is determined by the number and the average size of the original complexes predicted by an algorithm. If an algorithm prefers to predict a large number of complexes with small average size, the room for the improvement of *Sn* is very limited, since a very small number of new different complexes will be generated from the small original complexes. The number of complexes predicted by IPCA is the largest, and the average size is relatively small, thus the space to discard spurious proteins and identify more known complexes by the refining method is limited. If an algorithm tends to identify complexes with relative larger average size, there is great room for improvement, because more new different complexes will be reassembled by the refinement, such as Core-Attachment. As shown in Figure 6(b), the *Sp* of each algorithm after refinement is also boosted, and they are about 12% in CMC, 8% in CPM, 5% in DPClus, 6% in IPCA, 6% MCL, and 10% in Core-Attachment, respectively. It means although the number of refined complexes of most algorithms are increased, the percentages of *MPC* are also increased. The *f*-measure which is based on the increased *Sn* and *Sp* is also improved largely after refinement, and the average improvement is about 12%. The *f*-measure of each algorithms after refinement is enhanced by about 15% in CMC, 16% in CPM, 10% in DPClus, 8% in IPCA, 10% MCL, and 14% in Core-Attachment, respectively, as shown in Figure 6(c). These improvements validate the efficiency of the refining method, which can improve the ability of each algorithm to predict more *MKC* and *MPC*.

### Analysis of parameter *T*

**Figure 7:**
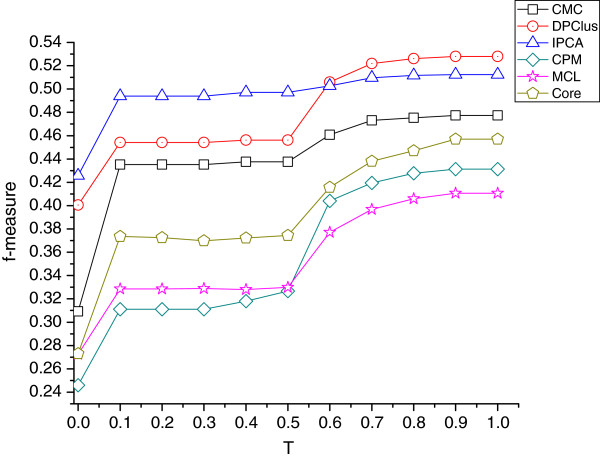
**The varying of *f *-measure of six algorithms under different *T * threshold.** Figure 7 shows how the different values of *T* threshold adopting in the refinement effect *f*-measure of six algorithms.

In the refining method, the value of *OV*’s threshold *T* is set as 0.6, while the average co-active rate in the known protein complexes is larger than 0.5. In this subsection, we will discuss the impact of different values of *T* on the prediction results. Actually, *T* can vary in the range of [0,1]. In Figure 7, the *f*-measure of each algorithms varies with different values of *T*. When *T*=0, the refined prediction results are the original ones. It is very easy to find out that, the *f*-measure of six algorithms are enhanced when *T*>0. We can observe that for all the six algorithms, when *T* is in the range of [0.1, 0.5], the changes of *f*-measure is insignificant. Because certain overlapping exists in the sub-clusters and is relatively easy to satisfy the combination condition when *T* is relatively small, the difference between the original ones and the refined ones is mainly the deletion of some proteins. The *PC* are not significantly increased, so are the *MPC* and *MKC*, which in turns have an influence on the *Sn*, *Sp* and *f*-measure. When *T* is in the range of (0.5, 1.0], the combination condition is more critical. It makes hard to combine sub-clusters, which makes the prediction more precisely. Thus *PC* is significantly increased, so are the *MPC* and *MKC*. For most algorithms, the *f*-measures are also significantly increased in this region, except for IPCA. As shown in Figure 7, the *f*-measure of IPCA after refinement is very flat in the range of [0.1, 1]. This is because the average size of the original complexes predicted by IPCA is relatively small, and the number of the original complexes is very large. On one had, it has tried its best to identify known complexes; On the other hand, the large number of original complexes with small average size limits the spaces to increase *MPC*, *MKC*, *Sn*, *Sp* and *f*-measure. Thus, for most algorithms, the value of *OV*’s threshold *T* is recommended in the range of [0.5, 1.0].

## Conclusion

Based on the just-in-time mechanism, the protein complex formation model is presented. Furthermore, we analyze the known protein complexes based on the complex formation model and combined with gene expression data, and find out that most complexes can be formed in a continuous time point set and the average overlapping rate of the known complexes during the formation is larger than 0.5. For the complexes which are predicted by clustering methods, only a small portion of them can be formed in a continuous time point set, and the average overlapping rates during the formation are significant lower than that of the known complexes. This paper proposes a method to refine the predicted complexes based on the protein activity and the complex formation model. The refining method contains two steps, splitting and assembling. To evaluate the refining method, we apply it to six algorithms which prefer to predict complexes with different sizes. Through the comparison of the *MKC*, *f*-measure, *Sn*, and *Sp* of original complexes and the refined ones, the results show that the *MKC*, *f*-measure, *Sn*, and *Sp* of each algorithm are significantly improved after refinement. Furthermore, it is easy to find out that the performance of algorithms which predict complexes with relative large average size has been greatly improved by our refinement method.

## Competing interests

The authors declare that they have no competing interests.

## Authors’ contributions

JXW and XQP obtained the protein-protein interaction data and gene expression data and designed the refinement method. XQP and QHX evaluated the results. JXW and XQP drafted the manuscript together. QHX, ML and YP participated in revising the draft. All authors have read and approved the manuscript.
